# End-stage kidney disease in patients with clinically manifest vascular disease; incidence and risk factors: results from the UCC-SMART cohort study

**DOI:** 10.1007/s40620-021-00996-1

**Published:** 2021-03-13

**Authors:** Helena Bleken Østergaard, Jan Westerink, Marianne C. Verhaar, Michiel L. Bots, Folkert W. Asselbergs, Gert J. de Borst, L. Jaap Kappelle, Frank L. J. Visseren, Joep van der Leeuw, the UCC-SMART studygroup

**Affiliations:** 1 Department of Vascular Medicine, University Medical Center Utrecht, Heidelberglaan 100, 3584 CX Utrecht, The Netherlands; 2 Department of Nephrology and Hypertension, University Medical Center Utrecht, Utrecht, The Netherlands; 3 Julius Center for Health Sciences and Primary Care, University Medical Center Utrecht, Utrecht University, Utrecht, The Netherlands; 4 Division Heart and Lungs, Department of Cardiology, University Medical Center Utrecht, Utrecht University, Utrecht, The Netherlands; 5 Faculty of Population Health Sciences, Institute of Cardiovascular Science, University College London, London, UK; 6 Health Data Research UK and Institute of Health Informatics, University College London, London, UK; 7 Department of Vascular Surgery, University Medical Center Utrecht, Utrecht, The Netherlands; 8 Department of Neurology, University Medical Center Utrecht, Utrecht, The Netherlands

**Keywords:** End-stage kidney disease, Modifiable risk factors, Incidence, Cardiovascular disease

## Abstract

**Background:**

Patients with cardiovascular disease (CVD) are at increased risk of end-stage kidney disease (ESKD). Insights into the incidence and role of modifiable risk factors for end-stage kidney disease may provide means for prevention in patients with cardiovascular disease.

**Methods:**

We included 8402 patients with stable cardiovascular disease. Incidence rates (IRs) for end-stage kidney disease were determined stratified according to vascular disease location. Cox proportional hazard models were used to assess the risk of end-stage kidney disease for the different determinants.

**Results:**

Sixty-five events were observed with a median follow-up of 8.6 years. The overall incidence rate of end-stage kidney disease was 0.9/1000 person-years. Patients with polyvascular disease had the highest incidence rate (1.8/1000 person-years). Smoking (Hazard ratio (HR) 1.87; 95% CI 1.10–3.19), type 2 diabetes (HR 1.81; 95% CI 1.05–3.14), higher systolic blood pressure (HR 1.37; 95% CI 1.24–1.52/10 mmHg), lower estimated glomerular filtration rate (eGFR) (HR 2.86; 95% CI 2.44–3.23/10 mL/min/1.73 m^2^) and higher urine albumin/creatinine ratio (uACR) (HR 1.19; 95% CI 1.15–1.23/10 mg/mmol) were independently associated with elevated risk of end-stage kidney disease. Body mass index (BMI), waist circumference, non-HDL-cholesterol and exercise were not independently associated with risk of end-stage kidney disease.

**Conclusions:**

Incidence of end-stage kidney disease in patients with cardiovascular disease varies according to vascular disease location. Several modifiable risk factors for end-stage kidney disease were identified in patients with cardiovascular disease. These findings highlight the potential of risk factor management in patients with manifest cardiovascular disease.

**Graphic abstract:**

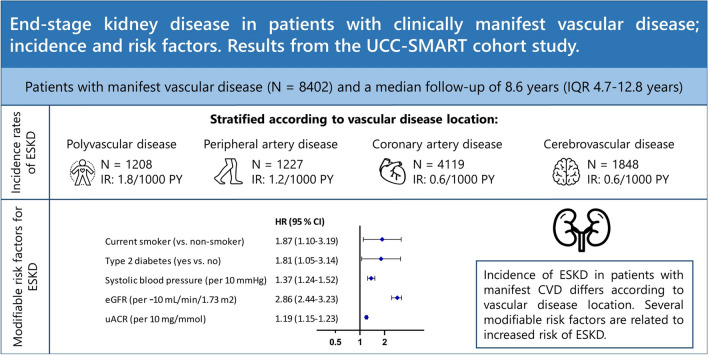

**Supplementary Information:**

The online version contains supplementary material available at 10.1007/s40620-021-00996-1.

## Introduction

Chronic kidney disease (CKD) is a growing health problem worldwide, predicted to be the 5th most common cause of life-years lost by 2040 [1]. The rise in CKD is mainly due to the increasing prevalence of type 2 diabetes mellitus (T2DM) and hypertension in the presence of increasing life expectancy [2]. CKD is irreversible and in most cases progressive, and the consequences include progression to end-stage kidney disease (ESKD), as well as an increased risk for cardiovascular disease (CVD) and mortality [3, 4]. The relation between CVD and CKD is bidirectional and patients with manifest CVD are at increased risk for adverse renal outcomes [5, 6].

Early identification and treatment of modifiable risk factors is the first-line strategy to reduce CKD progression in patients at high risk for developing ESKD, including patients with CVD at baseline. Known modifiable risk factors for ESKD include hypertension [7, 8], T2DM [9, 10] kidney function [11], obesity [12], dyslipidemia [13], smoking [14–16] and exercise [17]. However, these risk factors for ESKD are primarily investigated in low-risk populations and the effect of these risk factors may differ in patients with vascular disease, especially in more advanced cases. To the best of our knowledge, no previous study investigated the relation between modifiable risk factors for CVD and occurrence of ESKD in a high-risk population cohort with different manifestations of CVD, including cerebrovascular disease, coronary artery disease (CAD), peripheral artery disease (PAD) or polyvascular disease.

The aim of this study is twofold. First, we set out to determine the incidence of ESKD in patients with stable manifest CVD according to vascular disease location. The second aim was to assess the relation between modifiable risk factors for kidney disease and incident ESKD in a contemporary population cohort with stable manifest vascular disease.

## Materials and methods

### Study population

The study population consisted of patients included in the Utrecht Cardiovascular Cohort—Second Manifestations of Arterial Disease (UCC-SMART) study. The UCC-SMART study is an ongoing single-center prospective cohort study conducted in Utrecht, the Netherlands including patients from 18 years of age. A description of the study protocol has been provided elsewhere [18]. Study participants were patients newly referred to the University Medical Centre Utrecht with established CVD or an increased risk hereof, and were enrolled from September 1996 to February 2018. For this analysis, all patients with manifest cerebrovascular disease, CAD, symptomatic PAD and/or abdominal aortic aneurysm (AAA) were included. For definitions of CVD see Supplemental Table 1. Patients with ESKD at baseline were excluded (n = 20). The UCC-SMART study was approved by the local Medical Ethics Committee and written informed consent was obtained from all patients.

### Collection of data

All patients underwent vascular screening at baseline, including a health questionnaire, a standardized physical examination and collection of fasting blood samples. Estimated glomerular filtration rate (eGFR) was calculated using the Chronic Kidney Disease Epidemiology Collaboration (CKD-EPI) formula [19]. Systolic blood pressure (SBP) was measured three times on both arms with the patient in the supine position and the mean of the last two measurements of the highest arm was used. T2DM was defined as either a referral or self-reported diagnosis of T2DM, or a fasting plasma glucose ≥ 7 mmol/L at study inclusion with initiation of glucose-lowering treatment within 1 year, or baseline use of antihyperglycemic agents or insulin. Non-HDL-cholesterol was calculated as total cholesterol minus HDL-cholesterol, and LDL-cholesterol was calculated using the Friedewald formula up to triglyceride-values of 8.0 mmol/L. Smoking was self-reported and categorized as current smoking, former smoker or never smoker. Exercise was also self-reported as number of hours per week for sports, walking, cycling, and gardening, and this was multiplied by a specific metabolic equivalent of task (MET) derived from the Compendium of physical activity [20], resulting in a number of MET hours per week per activity. The total amount of physical activity was the sum of MET hours per week of all activities.

Participants were asked to fill out a questionnaire twice a year. If an event was reported, hospital discharge letters, relevant laboratory results and radiologic examinations were collected. With this additional information, all events were audited by three members of the UCC-SMART study endpoint committee, comprising physicians from various departments. The outcome of interest for this study was ESKD, defined according to Kidney Disease Improving Global Outcomes [21] as CKD stage 5 (eGFR < 15 mL/min/1.73 m^2^ not followed by any eGFR > 15 mL/min/1.73 m^2^), chronic dialysis or kidney transplantation.

### Data analyses

Data in the baseline table are presented as counts (percentages) for categorical values, as mean ± standard deviation (SD) for normally distributed variables and as median with interquartile range (IQR) for skewed distributions. The cohort was stratified according to previous vascular disease location. Vascular disease location was specified to either only cerebrovascular disease, only CAD, only PAD and/or AAA, or polyvascular disease defined as ≥ 2 locations.

To prevent loss of statistical power and potential bias [22], missing data were imputed by single regression imputation using all covariate and outcome data: eGFR (0.4%), urine albumin to creatinine ratio (uACR) (3%), smoking (0.4%), SBP (0.2%), body mass index (BMI) (0.2%), waist circumference (12%), non-HDL-cholesterol (0.6%) and exercise (23%). Incidence rates (IRs) and 95% confidence intervals (CI) were determined according to subgroups of vascular disease location. Kaplan–Meier survival curves were fitted to determine ESKD-free survival over time. To test for significant differences in ESKD-free survival between the groups, Peto’s log-rank test [23] was performed. In addition, survival curves based on an unadjusted Cox proportional hazard model was fitted with age at baseline and age at event as time-axis instead of follow-up time. This was done in order to illustrate the possible difference in life-expectancy free of ESKD between the subgroups of vascular disease location. The latter survival curve only included patients with a baseline age ≥ 50 years.

To assess the association between smoking, T2DM, SBP, BMI, waist circumference, non-HDL-cholesterol, eGFR, uACR and weekly exercise and ESKD, Cox proportional hazard models were constructed to determine hazard ratios (HRs) and 95% CIs. For eGFR as determinant, the inverse hazard ratio was determined (1/HR) in order to report risk of ESKD associated with decrease of eGFR. The linearity assumption between determinants and the log-hazard of ESKD was not violated based on visual inspection of restricted cubic splines. Satisfaction of the proportional-hazards assumption was confirmed by visual inspection of Schoenfeld residual plots. To adjust for confounding, three models were constructed: the first model was adjusted for sex and age and the second model was further adjusted for smoking, T2DM, SBP, BMI, non-HDL-cholesterol and exercise (if not a determinant of interest). A third model was constructed with addition of use of glucose-lowering medication, antihypertensive medication and lipid-lowering medication to the second model. All analyses were performed with R-statistic programming (version 3.5.1, R Foundation for Statistical Computing, Vienna, Austria). All p-values were two-sided, with statistical significance set at 0.05.

### Sensitivity analyses

Since eGFR and uACR are part of the causal pathway in the relation between determinants and risk of ESKD, we did not include them as confounders in the main analyses. However, since these markers of renal function may also partly act as confounders in the causal pathway, we performed analyses with these added to model 1. Also we show the hazard ratios of the crude data. Furthermore, for sensitivity analyses, the association between risk factors and ESKD was assessed in patients who were treated with RAS-inhibitors, as this is often used as treatment to prevent kidney function decline in high-risk patients and may thus act as an effect modifier in the relation between determinants and risk of ESKD. Also, as all-cause mortality constitutes a competing risk for ESKD, a Fine and Gray competing risk regression analysis was done with all-cause mortality as competing risk. Lastly, IRs were calculated stratified according to sex and age groups and interaction with sex and age, respectively, in the relation between determinants and risk of ESKD was examined.

## Results

### Baseline characteristics

A total of 8402 patients were included with a total follow-up of 75,131 person-years (median follow-up 8.6 years, IQR 4.7–12.8 years). Baseline characteristics of patients are shown in Table 1. Supplemental Table 2 shows the distribution of determinants and incidence rates for total mortality in patients who reached ESKD and in patients who did not. The mean age was 60 ± 10 years, 74% percent of the patients were male, 1848 (22%) had a history of only cerebrovascular disease, 4119 (49%) had a history of only CAD, 1227 (15%) had a history of only PAD and 1208 (14%) had a history of polyvascular disease. Patients with CAD or polyvascular disease were more often treated with antihypertensive and lipid-lowering medication. Patients with PAD were more often smokers and patients with PAD and polyvascular disease had on average higher SBP and lower levels of physical exercise. Patients with polyvascular disease had overall lower eGFR and higher uACR. Overall mortality risk during follow-up was 23% (IR 26/1000 person-years, 95% CI 25–27) and CVD risk was 19% (IR 22/1000 person-years, 95% CI 21–23).

**Table 1:** Baseline table

	Total (n = 8402)	Cerebrovascular disease (n = 1848)	Coronary artery disease (n = 4119)	Peripheral artery disease (n = 1227)	Polyvascular disease (n = 1208)
Gender, male [*n* (%)]	6199 (74%)	1059 (57%)	3341 (81%)	826 (67%)	973 (81%)
Age (years)	60 ± 10	58 ± 11	60 ± 10	59 ± 11	63 ± 9
Smoking, current [*n* (%)]	2561 (30%)	596 (32%)	930 (23%)	650 (53%)	385 (32%)
Type 2 diabetes [*n* (%)]	1386 (17%)	221 (12%)	718 (17%)	175 (14%)	272 (23%)
Physical exercise (MET h/week)	34 (17–63)	35 (18–61)	42 (22–70)	26 (10–51)	28 (11–52)
Systolic blood pressure (mmHg)	138 ± 21	140 ± 22	135 ± 19	143 ± 21	142 ± 21
Diastolic blood pressure (mmHg)	81 ± 11	82 ± 12	80 ± 11	83 ± 12	80 ± 12
Body mass index (kg/m^2^)	25.9 ± 4.1	25.4 ± 4.2	26.3 ± 3.8	25.0 ± 4.3	26.0 ± 4.0
Waist circumference (cm)	95.8 ± 11.8	92.6 ± 12.4	97.0 ± 11.3	94.6 ± 11.7	97.9 ± 11.7
HbA1c (%)	5.9 ± 0.8	5.7 ± 0.7	5.9 ± 0.8	6.0 ± 1.0	6.1 ± 1.0
Total cholesterol (mmol/L)	4.8 ± 1.2	5.0 ± 1.2	4.5 ± 1.1	5.5 ± 1.3	4.8 ± 1.1
LDL cholesterol (mmol/L)	2.8 ± 1.0	3.0 ± 1.1	2.6 ± 0.9	3.4 ± 1.1	2.8 ± 1.0
Non-HDL cholesterol (mmol/L)	3.6 ± 1.2	3.6 ± 1.2	3.3 ± 1.1	4.2 ± 1.3	3.7 ± 1.1
Triglycerides (mmol/L)	1.4 (1.0–2.0)	1.3 (0.9–1.8)	1.4 (1.0–1.9)	1.5 (1.1–2.2)	1.5 (1.1–2.2)
Serum creatinine (µmol/L)	87 (76–99)	83 (73–95)	88 (78–99)	85 (74–98)	92 (80–108)
eGFR (mL/min/1.73 m^2^)	77 ± 18	79 ± 18	78 ± 17	78 ± 19	71 ± 19
Albuminuria (micro) [*n* (%)]	1007 (12%)	204 (11%)	363 (9%)	199 (16%)	241 (20%)
Albuminuria (macro) [*n* (%)]	138 (1.6%)	23 (1.2%)	52 (1.3%)	33 (2.7%)	30 (2.5%)
Albumin/creatinine-ratio (mg/mmol)	2.6 ± 11.5	2.3 ± 9.7	2.0 ± 10.5	3.5 ± 14.3	4.2 ± 13.8
Use of antidiabetic medication [*n* (%)]	1129 (13%)	175 (9%)	601 (15%)	135 (11%)	218 (18%)
Use of insulin [*n* (%)]	378 (4%)	41 (2%)	203 (5%)	44 (4%)	90 (7%)
Use of antihypertensive medication [*n* (%)]	6297 (75%)	994 (54%)	3775 (92%)	543 (44%)	985 (82%)
Use of RASi medication [*n* (%)]	3579 (43%)	630 (34%)	1995 (48%)	343 (28%)	611 (51%)
Use of lipid-lowering medication [*n* (%)]	4508 (54%)	789 (43%)	2584 (63%)	415 (34%)	720 (60%)

*MET* metabolic equivalent of task, *eGFR* estimated glomerular filtration rate, *HbA1c* hemoglobin A1c, *LDL* low density lipoprotein, *HDL* high density lipoprotein, *RASi*  renin angiotensin system inhibition

### Incidence rates of ESKD according to vascular disease location

**Fig. 1:**
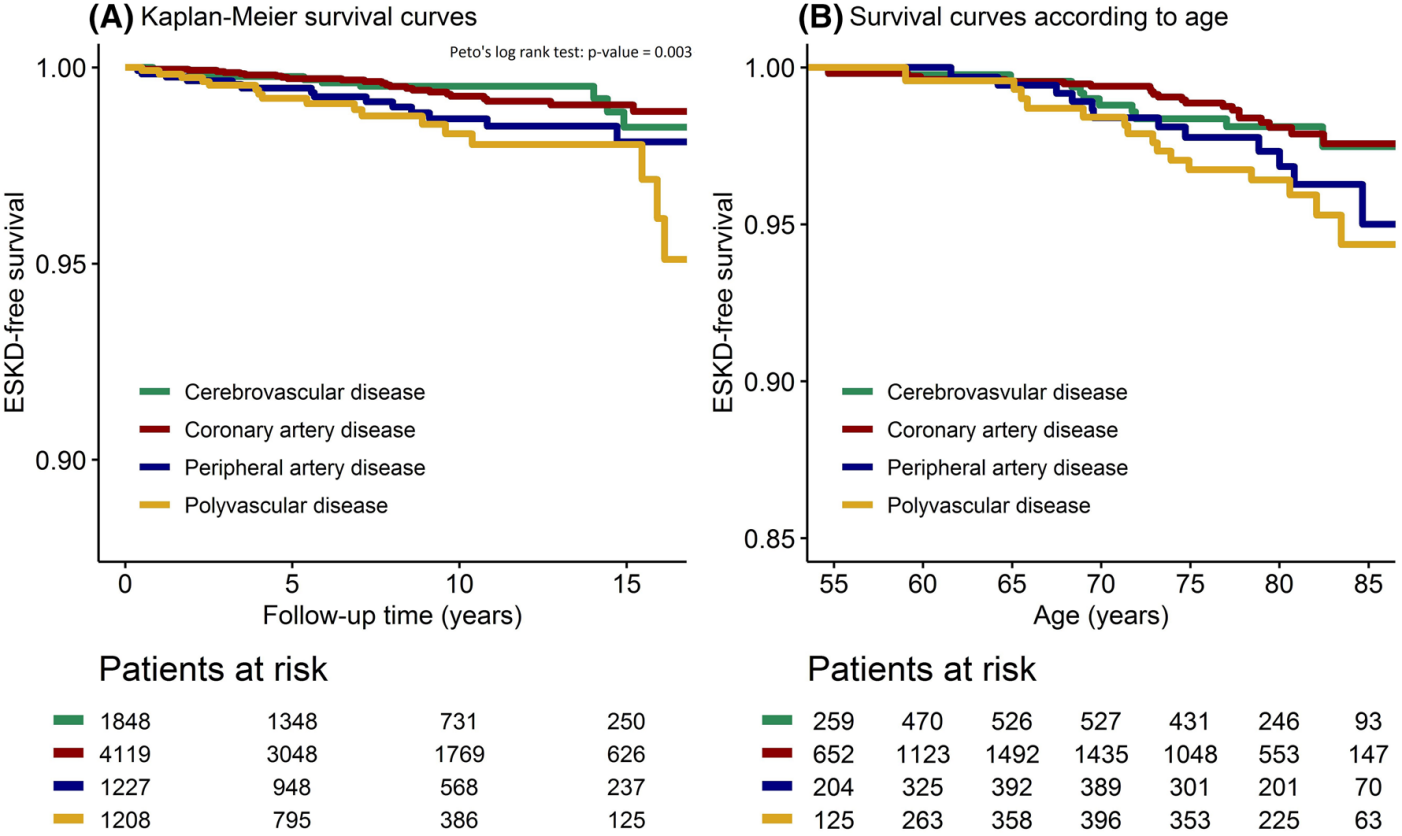
ESKD-free survival according to vascular disease location at baseline

A total of 65 ESKD-events were observed during follow-up (IR 0.9/1000 person-years, 95% CI 0.7–1.1). In patients with only cerebrovascular disease, 10 ESKD-events occurred (IR 0.6/1000 person-years, 95% CI 0.3–1.1). In patients with only CAD, 24 ESKD-events occurred (IR 0.6/1000 person-years, 95% CI 0.4–1.0). In patients with only PAD, 14 ESKD-events occurred (IR 1.2/1000 person-years, 95% CI 0.6–2.0) and in patients with polyvascular disease, 17 ESKD-events occurred (IR 1.8/1000 person-years, 95% CI 1.0–2.9). Overall absolute risk of ESKD was relatively small over time (Fig. 1a), and at the age of 50 years, patients with polyvascular disease had a shorter life-expectancy free of ESKD compared to patients with only cerebrovascular disease or only CAD (Fig. 1b).

### Relation between risk factors and risk of ESKD

**Fig. 2:**
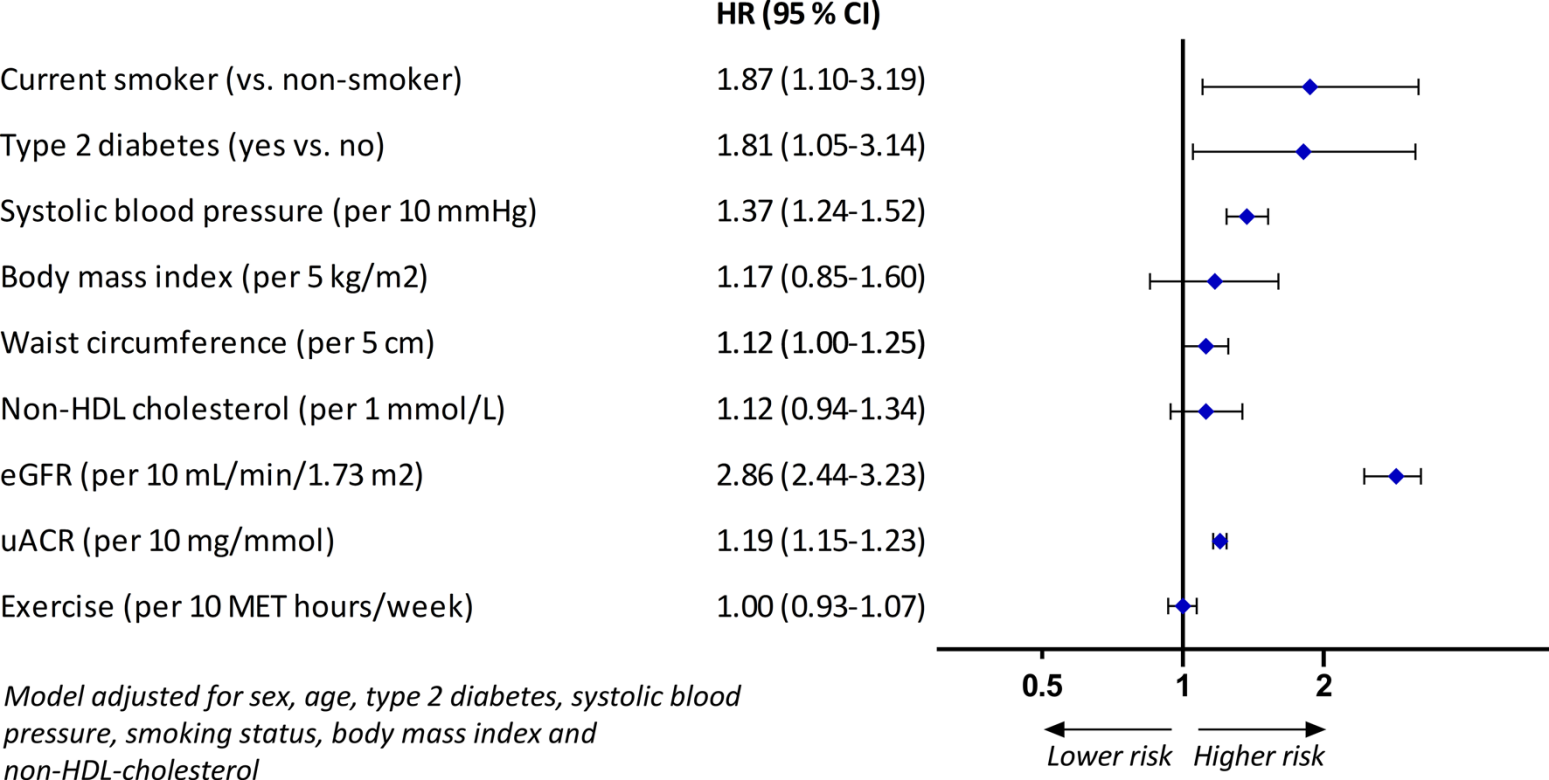
Relation between determinants and risk of ESKD

Using the model with clinical covariates, current smoking was independently associated with an elevated risk of ESKD (HR 1.87; 95% CI 1.10–3.19) and patients with T2DM had a higher risk of ESKD (HR 1.81; 95% CI 1.05–3.14). An increase in SBP was associated with an increase in the risk of ESKD (HR 1.37; 95% CI 1.24–1.52 per 10 mmHg). A 10 mL/min/1.73 m^2^ lower eGFR increased the risk of ESKD (HR 2.86; 95% CI 2.44–3.23) and a 10 mg/mmol higher uACR was significantly associated with higher risk of ESKD (HR 1.19; 95% CI 1.15–1.23) (Fig. 2). No significant independent relation was observed between physical exercise (HR 1.00; 95% CI 0.93–1.07), BMI (HR 1.16; 95% CI 0.85–1.60 per 5 kg/m^2^), waist circumference (1.12, 95% CI 1.00–1.25) and non-HDL-cholesterol (HR 1.12; 95% CI 0.94–1.34) and risk of ESKD. The magnitude and direction of the HR was not materially different compared with the model only adjusted for sex and age (Table 2), except that relations between non-HDL-cholesterol and risk of ESKD (HR 1.21; 95% CI 1.03–1.42) and waist circumference (HR 1.15, 95% CI 1.03–1.28) and risk of ESKD were significant. Further adjusting for use of medication added to the second model did not alter the HR meaningfully.

**Table 2:** Relation between determinants and risk of ESKD

	HR and 95% CI		
	Model 1	Model 2	Model 3
Current smoking (yes vs no)	1.90 (1.13–3.20)	1.87 (1.10–3.19)	1.93 (1.13–3.30)
Type 2 diabetes (yes vs no)	2.07 (1.21–3.54)	1.81 (1.05–3.14)	1.74 (1.00–3.01)
Systolic blood pressure (per 10 mmHg)	1.39 (1.26–1.54)	1.37 (1.24–1.52)	1.37 (1.24–1.51)
Body mass index (per 5 kg/m^2^)	1.23 (0.90–1.70)	1.16 (0.85–1.60)	1.12 (0.81–1.55)
Waist circumference (per 5 cm)	1.15 (1.03–1.28)	1.12 (1.00–1.25)	1.11 (0.99–1.24)
Non-HDL cholesterol (per mmol/L)	1.21 (1.03–1.42)	1.12 (0.94–1.34)	1.12 (0.96–1.30)
eGFR (per 10 mL/min/1.73 m^2^)	2.94 (2.50–3.33)	2.86 (2.44–3.23)	2.86 (2.44–3.33)
Albumin/creatinine-ratio (per 10 mg/mmol)	1.23 (1.19–1.27)	1.19 (1.15–1.23)	1.17 (1.13–1.22)
Exercise (per 10 MET h/week)	0.97 (0.91–1.04)	1.00 (0.93–1.07)	1.00 (0.93–1.07)

Model 1: Parameters included in the model are sex and age. Model 2: Parameters included in the model are sex, age, type 2 diabetes, systolic blood pressure, current smoking, body mass index, non-HDL cholesterol and exercise. Model 3: Parameters included in the model are sex, age, type 2 diabetes, systolic blood pressure, current smoking, body mass index, non-HDL cholesterol, exercise, anti-hypertensive medication, lipid-lowering medication and glucose-lowering medication (except for type 2 diabetes as determinant, which was not adjusted for use of glucose-lowering medication)

### Sensitivity analyses

Adjusting for eGFR and uACR did not meaningfully alter the direction of the hazard ratios, except for T2DM as determinant which became insignificant (Supplemental Table 3). When performing the analyses with all-cause mortality as a competing risk, the direction and magnitude of the hazard ratios did not change substantially (Supplemental Table 4). Furthermore, in patients who were using RAS-inhibitors, the hazard ratios for the relation between risk factors and ESKD also did not change considerably (Supplemental Table 5). IRs for ESKD were higher in men (1.0/1000 person-years) compared to women (0.5/1000 person-years) and in subjects older than 70 years of age compared to subjects younger than 70 years of age (Supplemental Table 6). However, no interaction between sex and age, respectively, and any of the determinants was observed (data not shown).

## Discussion

The present study shows that the incidence of ESKD in patients with stable manifest CVD varies according to vascular disease location. A higher incidence of ESKD and lower life expectancy free of ESKD was observed in patients with polyvascular disease or only PAD compared to patients with only cerebrovascular disease or only CAD. With respect to risk factors for ESKD in patients with stable manifest CVD, current smoking, T2DM, systolic hypertension, lower eGFR and higher uACR were all independently associated with increased risk of ESKD.

It is well known that the heart and kidneys are intertwined, in which dysfunction in one organ may induce dysfunction and increase the risk of disease in the other [3, 4]. The majority of previous studies examining the cardiorenal syndrome have focused on the relation between heart failure and CKD [24]. We expand on these previous findings by including patients with stable CVD with manifestations in different vascular beds.

The incidences of ESKD observed in the current study are higher than IRs reported in general population cohorts [11, 25, 26], indicating that patients with stable vascular disease have a higher risk of ESKD. A study performed in the CKD Prognosis Consortium cohorts found an IR for ESKD of 1.83/1000 person-years in populations with previous CVD or at increased risk of vascular disease [11]. A study examining the risk of ESKD after hospitalization with an incident CVD event reported an overall incidence of ESKD of 3.3/1000 person-years [27]. The incidence for ESKD in our study (overall IR of 0.9/1000 person-years) is lower, which might be due to the fact that the cohort consisted of patients who were overall intensively treated in terms of cardiovascular risk factors. Also, differences in case mix may strongly influence the incidence numbers across the studies.

In a broader perspective, approximately 1,550,000 people in the Netherlands are living with CVD [28]. Assuming the incidence rate found in this study, this will result in 1395 incident cases of ESKD per year. This agrees well with the incidence of ESKD-events within the Dutch population [29]. Since ESKD is associated with mortality and severe morbidity, reduced quality of life and increased health-care costs, this is a considerable number of events and focusing on the prevention of ESKD in high-risk patients with manifest CVD is important.

This study identified patients with PAD and polyvascular disease as patients at highest risk for ESKD. These findings may result from the identification of a population with more advanced general atherosclerosis, which also affects the aorta, renal arteries and the kidneys themselves, resulting in a higher risk of ESKD. The disparities in incidence of ESKD between men and women, with men having a higher IR than women, are complex and may relate to a faster decline of kidney function in men hypothesized to be related to protective hormonal effects in women and differences in lifestyle factors [30].

In the current study, several modifiable risk factors for ESKD in patients with stable CVD were identified. We observed a higher risk of ESKD in patients who were current smokers, patients with T2DM and patients with higher SBP. A previous study using general population cohorts found a relative risk for ESKD in subjects who were current smokers to be very similar to our results [15]. This underlines the importance of encouraging smoking cessation for both prevention of cardiovascular and kidney outcomes. Also, T2DM and SBP showed similar associations with ESKD as in the general population [8, 31], warranting close follow-up and treatment of these patients.

A previous meta-analysis found lower eGFR and higher uACR to be associated with increased risk of ESKD, regardless of traditional CVD risk factors [11], and albuminuria has previously been shown to be associated with increased risk of ESKD [32]. eGFR and albuminuria are measures of glomerular and tubular function and therefore intuitively important risk factors for ESKD. Also, a lower eGFR and higher uACR can both partly be attributed to the causal pathway between other risk factors and the development of ESKD. However, a lower eGFR is also associated with accumulation of uremic toxins, which increases progression of both CKD and CVD [33]. Specific treatment strategies, for example prescription of RAS-inhibitors [34], glucose lowering drugs [35] and lifestyle interventions [36], may alter this long term process by diminishing eGFR decline and reducing proteinuria. Increased awareness of these kidney function measures is likely to lead to better risk stratification and treatment in these high-risk patients.

Previous studies generally show obesity to be associated with increased risk of ESKD [12, 37–39], but little is known about the pathophysiology behind this relation. In the present study, larger waist circumference was found to be significantly associated with risk of ESKD when only adjusted for sex and age as confounders. A larger waist circumference is associated with higher insulin resistance [40], potentially leading to T2DM, which is a risk factor for ESKD. Thus, T2DM is likely part of the causal pathway in the relation between waist circumference and risk of ESKD. This was also suggested in our study, where the relation between waist circumference and risk of ESKD was slightly reduced when adjusting for T2DM. Furthermore, BMI was not found to be significantly associated with risk of ESKD. A recent study found a larger waist circumference to be associated with increased risk of ESKD, but no significant relation between BMI and risk of ESKD, as was also observed in the present study [41]. Since BMI is a composite measure of muscle- and bone mass as well as adipose tissue, waist circumference might be a more specific marker for adiposity. Also, as higher BMI is somewhat protective of CVD and ESKD in individuals at risk for malnutrition [42], such as people with advanced CKD or CVD, this might lead to reverse causality in the relation between BMI and risk of ESKD. These results indicate that obesity is a potential risk factor for ESKD in patients with manifest stable CVD, and waist circumference might be a better indicator for obesity when assessing this risk.

The major strengths of this prospective cohort study include the large number of patients with manifest CVD with extensive phenotyping of risk factors at baseline and a long and complete follow-up. Furthermore, the cohort is very contemporary as demonstrated by the high prevalence of preventive drug prescriptions. Also, the UCC-SMART cohort consists of patients referred with a broad spectrum of vascular diseases, making the results applicable to patients with various manifestations of CVD. Lastly, as patients with kidney disease often die of cardiovascular causes, we performed additional analyses to account for competing events and demonstrated similar results. Some limitations must also be considered. Baseline characteristics were only recorded at the start of the study but may have changed during the course of follow-up. Also, as ESKD develops over a longer time period there was a limited number of outcomes, thereby reducing the power of the study to find specific subgroup effects. Assessment of parameters known to influence vascular calcification, e.g. phosphate, calcium and serum levels of parathyroid hormone as risk factors for ESKD could also be relevant, but were unavailable in this study. However, their absence does not affect the validity of our findings.

In conclusion, the incidence of ESKD in patients with vascular disease is relatively low compared to vascular events and varies according to vascular disease location, being higher in patients with PAD or polyvascular disease. Modifiable risk factors for the development of ESKD in patients with stable CVD include current smoking, T2DM, systolic hypertension, low eGFR and high uACR. These findings highlight the potential of risk factor management in this high-risk patient group not only to prevent recurring vascular disease, but also to reduce progression to ESKD. This is in particular important when discussing risk factor management with patients and may enhance shared decision making by showing the importance of lifestyle changes and medication in the prevention of both recurrent CVD and ESKD.

## Supplementary Information

Below is the link to the electronic supplementary material.

Supplementary file1 (DOCX 23 KB)

## Data Availability

The data underlying this article cannot be shared publicly due to the privacy of individuals that participated in the study.
