# Supplementary material for: End-stage kidney disease in patients with clinically manifest vascular disease; incidence and risk factors: results from the UCC-SMART cohort study
Source: J Nephrol. 2021 Mar 13;34(5):1511–20. doi: 10.1007/s40620-021-00996-1 (PMC8494654; doi:10.1007/s40620-021-00996-1)
Supplement: Supplementary file 1 — Supplementary file1 (DOCX 23 KB) [file 40620_2021_996_MOESM1_ESM.docx]

**Supplemental material**

**Supplemental table 1. Definitions of cardiovascular disease**

| Cerebrovascular disease | A clinical diagnosis of a transient ischemic attack or ischemic or hemorrhagic stroke. |
| --- | --- |
| Coronary artery disease | A clinical diagnosis of angina pectoris, myocardial infarction, cardiac arrest or coronary revascularization. |
| Peripheral artery disease | Symptomatic and documented obstruction of distal arteries of the leg (ankle brachial index ≤ 0.90), a revascularization procedure of the leg (percutaneous transluminal angioplasty or bypass surgery) or a prior amputation. |
| Abdominal aortic aneurism | A history of abdominal aortic surgery or an abdominal aortic anteroposterior diameter of ≥ 3 cm at baseline. |

**Supplemental table 2. Distribution of determinants and incidence rates for total mortality in patients who did not reach ESKD and patients who did.**

|  | **ESKD outcome (n = 65)** | **No ESKD outcome (n = 8337)** |
| --- | --- | --- |
| **Current smoking** [n (%)] | 26 (40%) | 2535 (30%) |
| **Type 2 diabetes** [n (%)] | 19 (29%) | 1367 (16%) |
| **Systolic blood pressure** (mmHg) | 158 ± 23 | 139 ± 21 |
| **Body mass index** (kg/m^2^) | 27.3 ± 4.3 | 26.9 ± 4.0 |
| **Waist circumference** (cm) | 99.6 ± 11.4 | 95.8 ± 11.8 |
| **Non-HDL cholesterol** (mmol/L) | 4.0 ± 1.4 | 3.6 ± 1.2 |
| **eGFR** (mL/min/1.73 m^2^) | 45.7 ± 22.0 | 77.5 ± 17.4 |
| **Albumine/creatinine-ratio** (mg/mmol) | 27.4 ± 63.5 | 2.4 ± 9.9 |
| **Physical exercise** (MET hours/week) | 22 (7-55) | 35 (17-63) |
| **Incidence rate of mortality** | 83/1000 person-years | 26/1000 person-years |

**Supplemental table 3. Relation between determinants and risk of ESKD; crude data and markers of renal function included as confounders.**

| N = 8402, ESKD events = 65 | HR (95%CI) | |
| --- | --- | --- |
|  | **Model 1** | **Model 2** |
| Current smoking (yes vs no) | 1.41 (0.85-2.31) | 1.81 (1.06-3.10) |
| Type 2 diabetes (yes vs no) | 2.29 (1.34-3.91) | 1.47 (0.85-2.55) |
| Systolic blood pressure (per 10 mmHg) | 1.40 (1.28-1.53) | 1.21 (1.10-1.34) |
| Body mass index (per 5 kg/m^2^) | 1.17 (0.87-1.57) | 1.15 (0.84-1.58) |
| Waist circumference (per 5 cm) | 1.17 (1.05-1.30) | 1.15 (1.03-1.28) |
| Non-HDL cholesterol (mmol/L) | 1.16 (0.99-1.35) | 1.13 (0.93-1.39) |
| eGFR (per 10 mL/min/1.73 m^2^) | 2.79 (2.42-3.21) | 2.76 (2.38-3.19) |
| Albumine/creatinine-ratio (per 10 mg/mmol) | 1.21 (1.17-1.25) | 1.10 (1.06-1.14) |
| Physical exercise (per 10 MET hours/week) | 0.98 (0.92-1.05) | 1.03 (0.97-1.09) |

*Supplemental model 1: Crude data*

*Supplemental model 2: Parameters included in the model are sex, age, eGFR and albumine/creatinine ratio.*

**Supplemental table 4. Relation between determinants and ESKD in competing risk analyses with all-cause mortality as competing risk**

| ESKD outcome (n = 65) | Subdistribution HR (95%CI) |
| --- | --- |
| Current smoking | 1.67 (1.00-2.80) |
| Type 2 diabetes | 1.73 (1.01-2.95) |
| Systolic blood pressure (per 10 mmHg) | 1.36 (1.24-1.49) |
| Body mass index (per 5 kg/m^2^) | 1.16 (0.85-1.59) |
| Waist circumference (per 5 cm) | 1.11 (1.00-1.24) |
| Non-HDL cholesterol (mmol/L) | 1.12 (0.95-1.33) |
| eGFR (per 10 mL/min/1.73 m^2^) | 0.39 (0.33-0.46) |
| Albumine/creatinine-ratio (per 10 mg/mmol) | 1.16 (1.13-1.18) |
| Physical exercise (per 10 MET hours/week) | 1.00 (0.93-1.08) |

*Parameters included in the model are sex, age, type 2 diabetes, systolic blood pressure, smoking status, body mass index, non-HDL-cholesterol and exercise.*

**Supplemental table 5. Relation between determinants and risk of ESKD in patients treated with RAS-inhibitors**

| N = 3579, ESKD events = 39 | HR (95%CI) |
| --- | --- |
| Current smoking | 1.31 (0.64-2.68) |
| Type 2 diabetes | 1.75 (0.90-3.39) |
| Systolic blood pressure (per 10 mmHg) | 1.35 (1.20-1.53) |
| Body mass index (per 5 kg/m^2^) | 1.27 (0.87-1.86) |
| Waist circumference (per 5 cm) | 1.13 (0.99-1.29) |
| Non-HDL cholesterol (mmol/L) | 1.11 (0.84-1.45) |
| eGFR (per 10 mL/min/1.73 m^2^) | 0.35 (0.28-0.43) |
| Albumine/creatinine-ratio (per 10 mg/mmol) | 1.18 (1.13-1.23) |
| Physical exercise (per 10 MET hours/week) | 0.96 (0.88-1.06) |

*Parameters included in the model are sex, age, type 2 diabetes, systolic blood pressure, smoking status, body mass index, non-HDL-cholesterol and exercise.*

**Supplemental table 6. Incidence of ESKD stratified according to sex and age**

| Incidence rates per 1000 person-years (95% confidence intervals) | | | | | |
| --- | --- | --- | --- | --- | --- |
| Sex: | **Males** (n = 6199) | | **Females** (n = 2203) | | **P-value*** |
|  | 1.0 (0.7-1.3) | | 0.5 (0.3-1.0) | | 0.08 |
|  | | | | | |
| Age: | **< 50 years** (n = 1335) | **50-70 years** (n = 5635) | | **> 70 years** (n = 1432) |  |
|  | 0.6 (0.3-1.2) | 0.8 (0.6-1.1) | | 1.3 (0.7-2.3) | 0.10 |

**P-value is based on the Peto’s log rank test for testing difference between survival curves between the specific subgroups.*
